# Repositioning Natural Antioxidants for Therapeutic Applications in Tissue Engineering

**DOI:** 10.3390/bioengineering7030104

**Published:** 2020-09-02

**Authors:** Pasquale Marrazzo, Cian O’Leary

**Affiliations:** 1 Department for Life Quality Studies, Alma Mater Studiorum, University of Bologna, Corso d’Augusto 237, 47921 Rimini (RN), Italy; 2 School of Pharmacy and Biomolecular Sciences, Royal College of Surgeons in Ireland (RCSI), 123 St Stephen’s Green, 2 D02 Dublin, Ireland; cianoleary@rcsi.ie; 3 Science Foundation Ireland Advanced Materials and Bioengineering (AMBER) Centre, RCSI, 2 D02 Dublin, Ireland

**Keywords:** regenerative medicine, tissue engineering, anti-inflammatory, antimicrobials, biocompatibility, biomaterials, antioxidants, oxidative stress, tissue regeneration

## Abstract

Although a large panel of natural antioxidants demonstrate a protective effect in preventing cellular oxidative stress, their low bioavailability limits therapeutic activity at the targeted injury site. The importance to deliver drug or cells into oxidative microenvironments can be realized with the development of biocompatible redox-modulating materials. The incorporation of antioxidant compounds within implanted biomaterials should be able to retain the antioxidant activity, while also allowing graft survival and tissue recovery. This review summarizes the recent literature reporting the combined role of natural antioxidants with biomaterials. Our review highlights how such functionalization is a promising strategy in tissue engineering to improve the engraftment and promote tissue healing or regeneration.

## 1. Oxidative Stress in Tissue Engineering: The Rationale for Antioxidant Integration

Today, translation of tissue engineering research from the laboratory into the clinic continues to grow at pace, with applications ranging from aesthetic medicine to in situ biomedical sensors. Typically, clinical applications seek to repair or stimulate regeneration of degenerating or aging organs by surgical implantation of biocompatible biomaterials or cell-loaded biomaterials with the appropriate cues, as outlined in the principle of the “tissue engineering triad” [1]. This discipline is considered to hold a critical place in the future of widespread clinical practice, potentially forming a central role in treating an ageing population suffering from cardiovascular [2,3], musculoskeletal, periodontal [4], and diabetic conditions [5,6]. However, although promising in concept, the reality is that, in implementation, implanted tissue engineered constructs face obstacles that extend beyond the triad, with particular exposure to a stressed oxidative environment that can disrupt successful cellular repopulation and tissue regeneration after transplantation [7].

**Figure 1:**
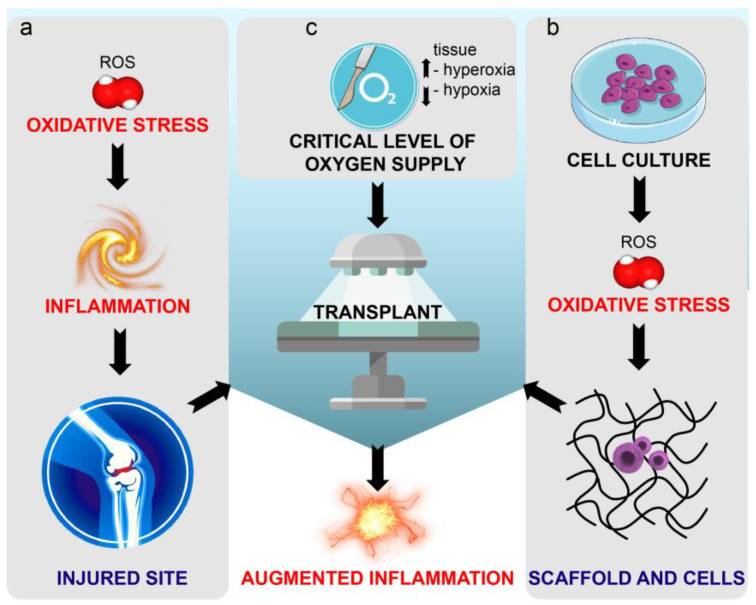
Schematic representation of the issues afflicting medical tissue engineering. (**a**) The injured site receiving the transplant is characterized by oxidative stress and pre-existing inflammation. (**b**) During cell cultures, the generation of reactive oxygen species (ROS) triggers harmful oxidative stress for cells included in biomaterial scaffold and intended for transplant. (**c**) During the transplantation, both the injury deriving from surgery and the oxygen supply can change tissue physiological oxygen levels. Taken together, the aforementioned conditions may augment inflammation and decrease the chance of success of tissue engineering.

As a consequence of injury or pathology, damaged tissue is exposed to a slew of biomolecules that are produced as the body attempts to repair itself. Among these molecules, free radicals and pro-oxidant species are generated by surrounding connective tissue and by immune responses. Although oxidative stress performs many physiological roles at low concentrations [8,9], it can be harmful in activity, depending on spatiotemporal factors such as the site, the amount of reactive oxygen species (ROS) generated, and their persistence over time. Accordingly, there is an increasing body of literature suggesting that the production of ROS and subsequent cellular response to oxidative stress are important for engraftment success [10]. Graft-versus-host disease (GVHD), for example, has been correlated to oxidative stress [11]. Indeed, tissue-engineered grafts are vulnerable to rising ROS levels (Figure 1), with reperfusion injury following ischemia reported in organ and tissue engineering transplantation [12,13]. Moreover, tissue oxygenation during in vitro graft preservation [14], along with its need during surgery, can cause a production of local hyperoxia and exacerbate ROS-related organ damage [15]. Taken together, these issues underscore the potential benefit that combating ROS with antioxidants in the implantation microenvironment can bring to limit post-implantation failure and tissue regeneration.

**Figure 2:**
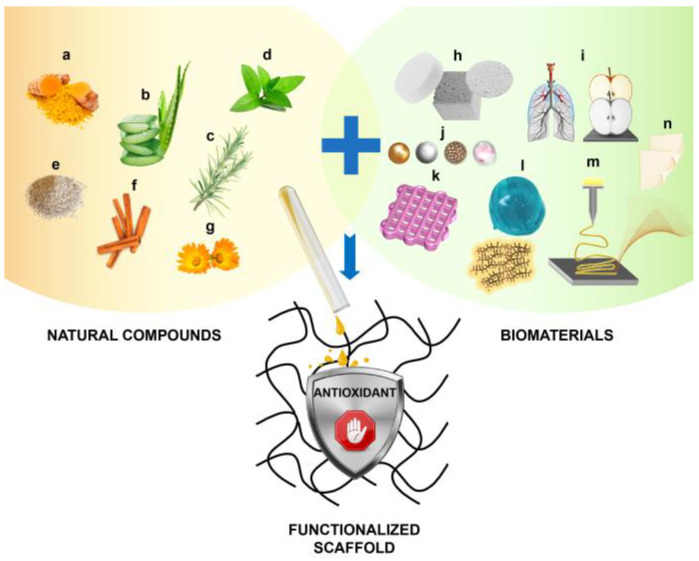
Schematic representation of antioxidant scaffold functionalization. Many natural sources (e.g., turmeric (**a**), aloe (**b**), thyme (**c**), green tea (**d**), sesame (**e**), cinnamon (**f**), and calendula (**g**)) are known to contain bioactive compounds with antioxidant effects that can be isolated and transferred to a scaffold for tissue engineering. Many biomaterials (e.g., ceramics and bioglasses (**h**), decellularized matrices (**i**), nanoparticles (**j**), bioinks (**k**), polymer and protein hydrogels (**l**), electrospun nanofibers (**m**), and dressing films (**n**)) are suitable for scaffold design and allow the association to such antioxidant compounds. The resulting scaffold will display new properties and should retain the compounds until it will be implanted.

Thus, a rationale has formed to consider the combination of anti-oxidant delivery with tissue engineering in order to improve outcomes in regeneration and restoration of organ function. Notably, an emerging trend is to reposition natural antioxidants to functionalize biomaterials with bioactive, ROS-reducing molecules (Figure 2). These biomolecules can regulate oxidation-reduction (redox)-mediated cell survival and homeostasis, whether for addressing redox balance in the in vivo microenvironment, or indeed for the more complex conditions of in vitro three-dimensional (3D) cell culture prior to surgical implantation. However, such functionalization is offset by the possible harm of total absence of ROS, which can stimulate initial healing processes. Moreover, the reactivity of antioxidants in their own right can pose stability concerns from a biomaterial perspective. Overall, in order to successfully reposition natural antioxidants into tissue engineering applications, it is critical to understand the essential concepts of antioxidants and how best to align them with tissue engineering principles. Accordingly, this review aims to provide such a context on core concepts and state-of-the-art. Firstly, an overview of antioxidants is outlined, followed by a summary of recent literature capturing the combined role of natural antioxidants with biomaterials, with a view to highlight how functionalization may be a promising strategy to improve the scaffold engraftment in tissue engineering.

## 2. Natural Antioxidants: An Overview

**Figure 3:**
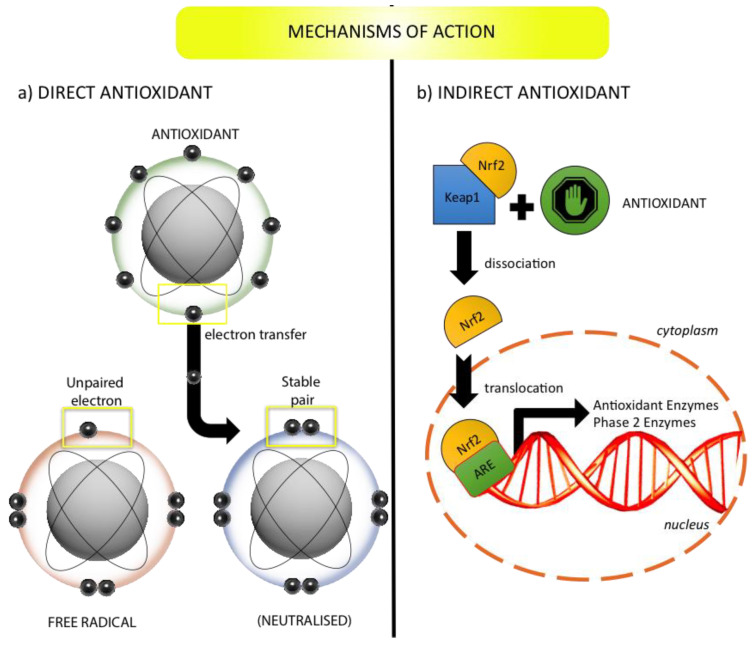
Graphical representation of the mechanism of action of direct and indirect antioxidants. (**a**) A direct antioxidant is redox active and short-lived. (**b**) An indirect antioxidant activates Kelch-like ECH-associated protein 1 (Keap1)/nuclear factor erythroid 2-related factor 2 (Nrf2)/antioxidant response element (ARE) pathway and augments endogenous cellular defences.

Antioxidants are defined as substances that, at low concentrations, could prevent the oxidation of a substrate [16]. They can be further divided in groups on the basis of their main mechanism of action (Figure 3) or on the basis of chemical structure. Direct antioxidants are chemicals that neutralize ROS through simple, direct reaction, and exert a classical scavenging activity. Indirect antioxidants are often activators of the Kelch-like ECH-associated protein 1 (Keap1)/nuclear factor erythroid 2-related factor 2 (Nrf2)/antioxidant response element (ARE) pathway, in which they induce phase 2 enzymes and augment cellular endogenous cytoprotective defences [17]. In comparison with a direct antioxidant that must be regenerated in order to continue its activity and can evoke a pro-oxidant effect, an indirect antioxidant may not be redox active and is unlikely to evoke a pro-oxidant effect [18]. Phenolic compounds are a large group of natural antioxidants. The polyphenols constitute a heterogenous sub-group of phenolic compounds, including flavonoids [19] and non-flavonoids. Flavonoids are sub-divided in flavan-3-ols (e.g., epigallocatechin gallate (EGCG)), flavonols (e.g., quercetin (Qu)), flavanones, flavones, isoflavones, and anthocyanins [20]. The non-flavonoid phenolic compounds are the proanthocyanidins (PA), the hydroxycinnamates [21] (e.g., caffeic acid, rosmarinic acid (RA), ferulic acid, and cinnamic acid), the lignans (e.g., sesamol), the stilbenes (e.g., resveratrol), benzoic acids (e.g., ellagic acid), coumarins (e.g., osthole), and curminoids (e.g., curcumin (Cur)) [22]. Terpenoids (e.g., carotenoids, thymol) are also common dietary antioxidants, usually occurring alongside polyphenols [20]. Other bioactive phytochemicals are nitrogen-containing compounds (e.g., isothioacyanates) or sulfur-containing ones [20]. Some vitamins, like vitamin C and E, are essential nutrients as well as molecules displaying an important antioxidant effect. 

Conventionally, antioxidant sources have been largely investigated in terms of their scavenging potential of oxidant species; however, in the last decades, increasing attention has been directed to demonstrate a natural antioxidant protective effect on cells and organs. Principally, the polyphenols naturally biosynthesized by plants and marine organisms are well known as antioxidant and protective compounds. Several biochemical pathways, including regulating nuclear transcription factors, have been associated especially with polyphenols to promote health benefits [20]. Indeed, natural antioxidants raise attention because of their several capacities, that is, to induce the stress-response pathway activated by the Nrf-2 transcription factor [23] or inhibit nuclear factor kappa-B (NF-kB)-mediated inflammation pathway [24] or decrease the NADPH oxidase (NOX) family effect [25].

One disadvantage of dietary antioxidants is their bioavailability and stability. In fact, antioxidants, like their opposite ROS, are highly reactive [26], making it difficult to store them without preventing accidental oxidation. In the same manner, even after the in vivo delivery, their reactivity can result in low bioavailability. Two hypotheses are proposed for reduced bioavailability. First, in the case of oral administration, some natural antioxidants are activated only after enzymatic cleavage by human microbiota. The bioavailability of antioxidants following the dietary intake depends on digestion, then on gut absorption, metabolism, and clearance. Second, their active form is not feasible to be delivered after the implantation to the specific tissue target because of its intrinsic physicochemical properties. One potential solution to explore to overcome the instability related to a single antioxidant is to consider the use of multiple antioxidants together in a complex network. Indeed, recently in vitro results have suggested that a combination of antioxidants synergizes their effect in terms of cytoprotection [27], regeneration [28] and healing [29]. Such a consideration supports a similar co-delivery strategy for medical tissue engineering purposes. To date, the effects of the antioxidants in available human clinical studies were not significant and have some limitations [30], including the lack of clinical biomarkers and monitoring of oxidative load for effectiveness of the prescribed antioxidant [30]. Although the current antioxidants appear unsuccessful in the treatment of cardiovascular diseases [31], antioxidant strategies still represent an avenue of treatment for cardiovascular disease [31]. Similar disappointing clinical outcomes were reported for disease-modifying therapies for stroke [32] or in treating chronic neurodegenerative diseases [33]. The limitations for single antioxidant treatment in chronic neurodegenerative conditions can belong to insufficient knowledge about the biological equilibrium that can maintain a proper level of oxidative activity as well as a “chemical failure” due to poor blood–brain barrier permeability [32]. Unfortunately, most trials for vitamins were done in patients with established pathology, but preventing the disease could be a more realistic expectation than abolishing symptoms [30].

## 3. Biomaterials: Overview of Natural Polymers 

**Figure 4:**
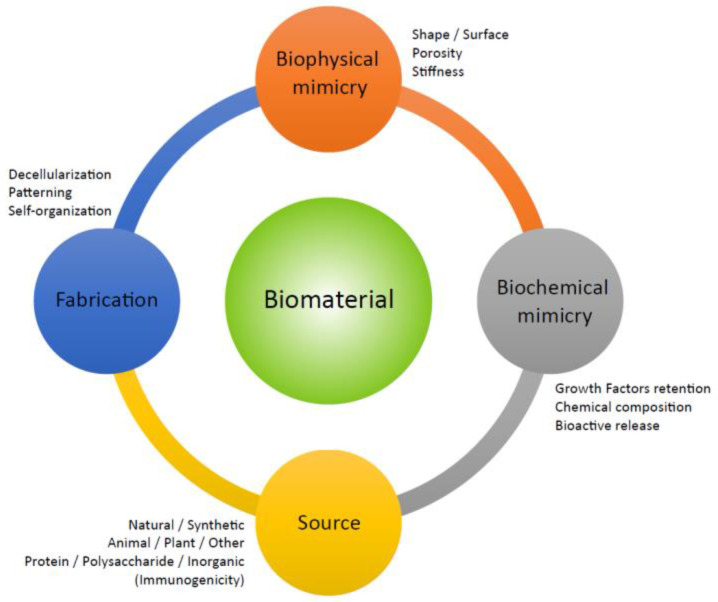
Schematic representation of the criteria to consider during biomaterial design. The properties of the biomaterial will be dependent on both biophysical and biochemical cues, strictly connected to the source of the material and its fabrication methods.

When considering the design of a biomaterial scaffold (Figure 4), naturally-derived materials that resemble native tissue architecture can offer clear advantages [34]. Decellularized extracellular matrix (ECM) understandably [34] displays the closest approximation to native tissue. Decellularized matrix can be obtained both by animals and plants and protein matrix isolated can retain cell cues and growth factors (GFs). Animal-free matrices are very useful for 3D bioprinting of grafts [35] and decellularized matrices are ideal materials for generating a bioink [35]. As animal-derived matrices have to deal with xenogenic and ethical issues [36], plant-derived matrices are a worthy option. Matrices obtainable via plant tissue decellularization [37] are extremely challenging in the tissue engineering field and scaffolding for 3D cell cultures, however, they could need smart functionalization because of some lack of biochemical mammalian cues [38]. Strategically, plant-derived scaffolds and decellularized matrices [39] are biocompatible [40], bioprintable [41], and suitable to induce human stem cell differentiation [42] like osteogenesis from induced pluripotent stem cells (iPSCs). Polysaccharides, another class of natural biomaterial, are feasible polymers to develop ideal non-immunogenic scaffolds, usually in the form of hydrogels [43]. A critical review of all the possible interactions of polysaccharide biomaterial with the immune system [44] highlighted the need for standardization protocols for the natural polysaccharides and that the components of a composites could differentially produce unknown responses. Polysaccharides [45] often have good biocompatibility and are eco-compatible with a sustainable industry [39]. Polymers are attractive as future antioxidant materials, as many of them derive from plant sources [46]. Indeed, instead of biomaterial functionalization by antioxidants, there are hydrogels made of polyphenolic antioxidants in their own right. *Aloe vera* (AV) is the most known species of *Aloe* genus. AV is both a rich source of phytochemicals and an exciting natural scaffold for tissue engineering [47]. Its clinical efficacy was demonstrated [48]; indeed, it is used in topical dermatology applications; in cosmetics; or in wound healing as an anti-inflammatory, immunomodulatory [49] and antiseptic/antimicrobial agent [50]. Similar to cellulose, the lignins from forestry biomass can complex with synthetic polymers, for example, polylactic acid (PLA), to fabricate scavenging antioxidant material by [51] printing. Natural gums, namely marine alginate (ALG) [52], or plant-derived gum like arabic gum, can provide structural compatibility to the in vivo architecture, and thus are attractive biomaterials for innovative tissue engineering strategies [53]. Therefore, polymer scaffolds can derive from naturally agricultural products and byproducts [37], implementing the panel of techniques available for in 3D in vitro culture of mammalian cells. This could be a sustainable approach towards the next decade of biomedical needs. Other popular natural biomaterials include silk and chitosan.

Silk fibroin (SF) is a structural fibrous protein obtained from the cocoon of *Bombyx Mori* and it is also suitable for electrospinning [54]. Indeed, SF use is abundant to design porous scaffold, especially nanofibers [55] for bone [56] and cartilage [57] tissue engineering. Chitosan (CH) is a well-known, semisynthetic biomaterial [58] derivative of chitin [59,60] that has similarities to cellulose in its structure. Chitosan is an outstanding scaffold [61] because it is sustainable [62] and exerts antimicrobial [63] and immunomodulatory properties [64]. Not only does it possess intrinsic antioxidant capacity [65], but chitosan is also a good candidate for drug delivery [66], tissue engineering [67], and pharmaceutical engineering [67], and thus open to vast functionalization.

Thus far, biomaterials designed from metals, ceramics, and recently bioglasses [68] mimic particular biomechanical features (e.g., for bone and enamel). They are usually divided into inert or bioactive. The inert materials have been a spread use in the past for prosthetic implants where a mechanical support was the primary sufficient and requested activity for the biomaterial.

## 4. New Opportunities for Natural Antioxidant Combination with Tissue Engineering

### 4.1. Oxidative Stress Reduction Ex Vivo and In Vivo

**Figure 5:**
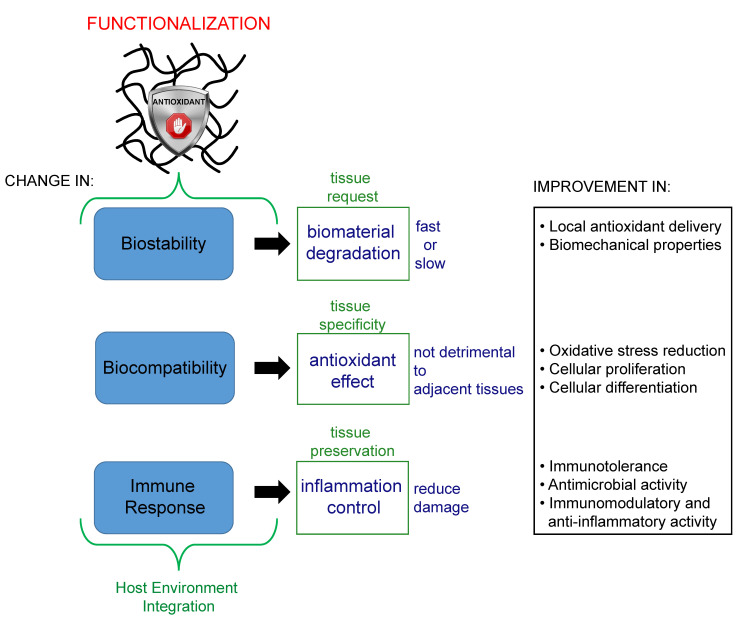
Schematic representation of the properties of a biomaterial that are influenced by antioxidant functionalization.

Essentially, the antioxidant potential of bioactive compounds, together with their health protective effects, could represent a promising tool in regenerative medicine. The studies showing antioxidant activities mediated by different functionalized biomaterials are listed in Table 1. Antioxidant solutions have been tested or used to slow ex vivo organ degeneration [10] before transplantation, and research is investigating alternative antioxidant buffers for the preservation of ex vivo biological material viability. In a similar manner, antioxidant supply could preserve ex vivo graft survival, such as cornea and skin graft that, in the last decades, have been used at good manufacturing practices (GMP) level for clinical application [69,70]. As all tissues share many basic mechanisms to maintain a redox balance, the modulation of oxidant species levels by antioxidants can be widely applicable. Basically, the introduction of an antioxidant in tissue replacements will cause an effect depending on released concentration and ECM interactions. Finally, it could result in scavenging activity of radicals or in endogenous defence enhancement. Endogenous antioxidant molecules may cooperate with exogenous antioxidants to decrease the chance of failure of the graft, as well as they will promote a safe fate of both exogenous and endogenous cells connected to the implanted biomaterials. More appreciably, antioxidant supply can avoid biomaterial-mediated increase of oxidative stress after implantation. In respect to tissue oxygenation, the delivery of antioxidants could avoid excessive tissue oxygen levels that may lead to ROS production and subsequent dysfunction in the transplanted graft. “Tuneable” activation of antioxidant biomaterials is highly desirable [71]. Indeed, sensing before being responsive is a need during relevant increase in oxidative stress. Such a kind of activation is ideal for clinical application of antioxidant biomaterials [72]. With the goal of reducing oxidative stress, new biomaterials can be exploitable in order to control the release of redox sensitive molecules, that is, disulphide containing biomaterials [73]. Moreover, safeguarding redox equilibrium is also critical in tissue engineering for angiogenesis [74], an essential step promoting long-term survival and engraftment. In summary, antioxidants can preserve the viability of transplantable cells before and after transplantation, as well as control the oxidative stress in the microenvironment of implanted biomaterials (Figure 5).

### 4.2. Local Delivery

Locally targeted therapy is a must in a clinical setting to avoid organ toxicity and side-effects. For biomaterials, their direct association with specific growth factors can involve and activate many pathways, however, leading to specific different effects on different cell types via cell receptors. In contrast, physiological concentrations of antioxidants whose delivery is restricted to host tissue adjacent to the biomaterial will possibly have a broad effect on redox homeostasis, with a mechanism broadly shared among all cells within the body. Implants and grafts are often at the boundaries among different tissues, thus the functional molecules coupled to the biomaterials should not be detrimental to the close tissues. Furthermore, the effect of the antioxidant should be active specifically towards target tissue. The development of functionalised biomaterials [75] specifically retaining different natural bioactive compounds can contribute to a new rational delivery of antioxidants, with the aim of reducing oxidative stress and their downstream risk to aggravate a disease in the human body. Antioxidants can augment the potential of such biomaterials through local delivery. They can be introduced in biomaterials together with their biochemical limitations. Antioxidant nutraceutical compounds are hydrophilic or lipophilic molecules. In addition, active compounds also showed different cellular distribution. The inclusion of the antioxidant into the biomaterial via coating or during scaffold synthesis, in respect to other delivery routes, could reduce the required administered amount. This can be helpful for antioxidants as well as pharmaceutical compounds with low systemic stability or diffusion to the surrounding anastomosis. Thanks to its polysaccharide content and its absorption capability, AV, for example, can deliver drugs as well as additional dietary micronutrients and increase their local bioavailability [76]. The physical form and the formulation of the associated biomaterial could modulate the delivery of the antioxidant as well as solve some of its application limits, for example, encapsulation of carotenoids in nanocarriers can counteract their poor bioavailability and stability [77]. Recently, nanoparticles have received increasing attention as a tool to deliver the most different bioactive agents [78]. Depending on the application, different organic and inorganic materials and their composites can be developed for nanoparticles design. Gold nanoparticles, for example, are useful deliver a large set of molecules, including natural antioxidants. The nanoparticle seems the most used form of carrier for natural compound drug delivery, however, naturally-derived hydrogels [79] could be highly suitable for the same use. Overall, an antioxidant should be coupled to the biomaterial to achieve different goals in timeline, that is, to reach the specific tissues, to be locally available at effective physiological concentration while avoiding non-specific effects (Figure 5). The studies showing antioxidant delivery control mediated by different functionalized biomaterials are listed in Table 1.

### 4.3. Biodegradability and Biomechanical Properties

Biomaterials for in vivo application, as injection and implant, need to exhibit critical degradation properties. Biodegradability is an essential process as new tissue forms during healing, similar to the native one and without artificial elements. On the other hand, some biomaterials require a long-lasting duration into tissues or permanent integration into them. For materials functionalized with antioxidants, controlled release of non-covalently bonded antioxidant can be achieved less or more slowly, in balance with the scaffold’s physical features and biodegradation. Regarding biomaterial mechanical integrity, protein cross-linker agents offer degradation resistance to natural scaffolding biomaterials, but chemical agents such as glutaraldehyde are usually less tolerated and toxic. Alternatively, the inclusion of antioxidants can be obtained by adsorbing, trapping them within the scaffold or by previously incorporating them in a co-formulation (e.g., copolymer, solution). According to the planned delivery of the antioxidants, a different chemistry will be selected for the inclusion. Even surface modification of biomaterial [80], especially for non-protein biomaterial, is an option to provide the tissues of antioxidants. Indeed, a coating as a source of antioxidant has the advantage that it can be associated with both artificial and nature-inspired scaffold material. Three-dimensional bioprinting has become a tremendous field of research and bioink development is a tool for challenging clinical application [81]. For 3D printing of photocrosslinkable biomaterials, some efforts are still necessary to obtain optimal biological functions and mechanical integrity [82].

Many antioxidants are useful to improve the scaffolding ability of the biomaterial, in particular, polyphenols are used as modular building blocks for the engineering of materials for biological application [83]. Their physicochemical configuration, including hydrogen bond formation, metal coordination, amphiphilic nature, and sensitivity to oxidation, makes them suitable to adhere to various substrates. Then, polyphenols can assemble in intelligent nanoparticles, films, and gels to shape the biomaterial. Polyphenols are agents used in the preparation of biomaterials and are a modulator of biomaterial function. Therefore, the antioxidant functionality of the biomaterial can be designed by selecting polyphenols according to the number and binding structure of phenol units. As a result, one main advantage of coupling biomaterials to natural antioxidants would be an increased durability of the modified scaffolds. The preservation of matrix proteins that can be degraded by inflammation and consecutive extracellular pH changes [84,85,86] is particularly important for biomaterials, which are prepared as aminoacidic/protein hydrogel or decellularization techniques. Collagen is the major fibrous extracellular matrix protein, abundant and biomechanically important in the body. The collagen fibrils are present in differentiated connective tissues like tendons and pericardium, widely present in vessel sub-endothelial structures, cartilage surfaces, bone matrix and dentin. Like collagen, elastin is a structural protein making the network of fibers in the extracellular matrix (ECM). Moreover, elastin is not stabilised by glutaraldehyde; therefore, other molecules such as some phytochemicals can similarly or even better improve the physical integrity of protein and polymeric biomaterial. Notably, a large number of polyphenols (e.g., pentagalloyl glucose) are able to enhance the stability as well as the strength of collagen or elastin fibres with different mechanisms [87]. The tannins, in particular the class of PA, are flavonoids with a great influence on biomechanical properties of both collagen and elastin. In general, several studies reported that collagen and elastin express high affinity for PA because of their intrinsic abundance of proline. The interaction via covalent or hydrogen bonds of those flavonoids improves the stability and strength of extra cellular matrix fibres, in general by increasing denaturing temperature and inhibiting proteolytic enzymes such as collagenase and elastase matrix-metalloproteases (MMP). Other natural molecules are spreading as eco-friendly cross-linking agents; genipin is an aglycone iridoid usually isolated from extracts of gardenia fruits. Genipin is a non-toxic “green” cross-linker [88,89], used to build scaffolds tested in 3D in vitro cell culture [90,91], and exhibiting anti-inflammatory [92] antibacterial [93] and antiglicative activities [94]. Metals and other inorganic materials are also used in protocols for biomaterial engineering. Some metal biomaterials including antioxidants were built for tissue engineering studies. For instance, gold nanoparticles are a tool in tissue engineering [95] to ameliorate mechanical properties of the biomaterial and to guide specific cell behaviour [96]. Resveratrol is a feasible reducing agent for the synthesis of gold nanoparticles avoiding self-agglomeration and enabling additional drug loading [97]. Epigallocatechin gallate (EGCG) was used as linking agent between collagen fibres and palladium nanoparticles [98] in order to stabilize the metal nanoparticles. The capacity of antioxidants to modify physical properties of a specific biomaterial (e.g., wettability, permeability) could also be revolutionary for development of theranostic nanomaterials [9]. In conclusion, antioxidant functionalization not only theoretically provides antioxidant effects, but also can increase the structural stability and modify the degradation profile (Figure 5). The studies showing physico-chemical property change mediated by different functionalized biomaterials are listed in Table 1.

### 4.4. Biocompatibility

For clinical application, a biomaterial possesses a combination of advantageous properties, including biocompatibility and non-toxic characteristics, for example, supporting cell adhesion, proliferation, specific cell differentiation, and host immune system tolerance or immunomodulation (Figure 5). In the design of a biomaterial, the diffusion of nutrients and waste must be facilitated. The efforts on topology can be performed for optimization and establishment of both biochemical signals and cell polarity. Natural materials have inherent biochemical cues [99] that better allow cell attachment and differentiation. A critical factor in the cell–biomaterial interaction is the hydrophilicity of the scaffold [100]. The biomaterial surface provides suitable physical contacts to the cells. The wettability influences not only the cell adhesion, but also successive proliferation or differentiation [100]. For this reason, biocompatibility is highly influenced by the physicochemical properties of the biomaterial (par. 4.1–3). Importantly, a specific antioxidant molecule should be selected on the basis of the level of stimulation on the cells of the specific tissue target, such as induction of proliferation or differentiation. After the selection of the antioxidant, different endpoints for toxicology assessment can be indicated by in vitro testing [101], in general, cytotoxicity, cytokine release, and oxidative stress [102], as well as embryotoxicity and epithelial barrier integrity, according to the tissue origin. Finally, assessing in vitro and in vivo biocompatibility is an essential requirement before the designed biomaterials with gained antioxidant potential would be applied in vivo, in new different tissue environments. The studies showing biocompatibility as well as cellular proliferation and differentiation mediated by different functionalized biomaterials are listed in Table 1.

### 4.5. Immunomodulation and Pathogen Defense

The immune system is the key orchestrator in the regenerative response, independent of the allograft or autograft properties. Unfortunately, immune reaction against the biomaterial and downstream unproductive inflammation are the main causes of failure for several regenerative strategies based on scaffold graft. It is not rare that novel biomaterials, including biopolymers, which display biocompatibility and support cell viability, are also highly immunogenic or stimulate an inflammatory reaction [44]. This inflammatory reaction, as well as the final healing, is linked with changes in redox equilibrium. Oxidative stress derives from both pre-existing inflammation condition in the host tissues and from the usual surgical wound. Particularly, the chemokines previously and continuously produced by the recruited immune cells, the temporary hypoxia, the protein adsorption to the biomaterial, derived from blood elements (e.g., platelets, complement) and the interstitial fluid consequent to the implantation, and muscle and red blood cell lysis at wound boundaries could contribute to excessive and injurious generation of ROS. Hydrogen peroxide, released by neutrophils to reduce bacterial infections [103], recruits phagocytic leukocytes, together with factors secreted by activated mast cells [104]. Besides the ROS burst, neutrophils degrade the biomaterials via production of proteolytic enzymes. The activation by ROS of M1 macrophages and their persistence in the peri-implant site together with the formation of foreign-body giant cells forwards the chronic inflammation instauration. Positively, ROS are able to modulate macrophage polarization towards M2 phenotype, as the inhibition of ROS production specifically affects the polarization to M2 and not to M1 phenotype [105].

Remarkably, natural antioxidant inclusion in a promising biomaterial can both improve biocompatibility of the scaffolds and favour a temporary immunological tolerance towards it. To allow new tissue formation and control inflammation rather than amplify the deleterious side effect induced by ROS, natural antioxidants can appropriately be associated to the scaffold to immunosuppress and regulate the inflammatory response (Figure 5) by different cell types. Natural antioxidants such as phenolic compounds have the advantages to enhance immunocompetence [103,106] and to be low toxic for human cells [107]. More importantly, multiple compounds isolated from plants and fungi received much attention in experimental in vitro research because they modulate endogenous antioxidant systems rather than simply neutralizing free radicals. Antimicrobial activity is often associated with many antioxidants. Indeed, plants and other organisms produce antimicrobial antioxidant to protect themselves from the attack of parasite or pathogens [108]. It is important to highlight that bacterial infection is a significant risk with bio-engineered tissues, so the antimicrobial activity of the antioxidant constitutes an important feature in the context of clinical engraftment. Indeed, avoiding opportunistic infection following human transplantation and wound healing is an essential point for the clinical outcome of the patient receiving a particular biomaterial, in order to restore tissue functionality. Metal biomaterials use is strictly related to an intrinsic anti-bacterial capacity. So far, silver particles were used as common agents to produce antibacterial effect within the biomaterials [78], but recently, silver biomaterials also evolved into an antioxidant carrier. In brief, naturally occurring antioxidants are expected to be included into new biomaterial production and future medical devices for clinical application in which the immune system plays a key role. The studies showing antimicrobial, immunomodulatory, and anti-inflammatory effects mediated by different functionalized biomaterials are listed in Table 1.

The introduction of the antioxidant into the biomaterial may change its biostability, biocompatibility, and the immune response by the host. In particular, the degradation of the biomaterial, the effect of the antioxidant, and the inflammation control by the functionalized biomaterial are a result of the host tissue integration. The properties should be in accordance with tissue needs.

## 5. Towards Therapeutic Application: Antioxidant Biomaterials under Investigation

### 5.1. Wound Healing

Inflammation is a necessary component of healing in skin and mucosal wound healing [229]. In healing, there is typically an initial inflammatory phase, then a proliferative phase, and finally tissue remodelling; all the variations in these phases depend on the biomaterial features, but also on inflammation status sustained by oxidative stress at the injury site. Wound healing studies dominate the range of the applications of biomaterials investigating natural antioxidants. This popularity is likely owing to the fact that many herbal compounds were traditionally utilized as wound healing agents.

Antioxidants have been incorporated into a range of synthetic biomaterial polymers. Studies with the antioxidant curcumin (Cur) typify this line of research. Polylactic acid (PLA) and hyperbranched polyglycerol (HPG) blend was used to produce nanofibers loaded with Cur that demonstrated high swelling and great in vitro biocompatibility for 3T3 fibroblast cells. The presence of hydrophilic and non-immunogenic HPG polymer was critical to complete the in vitro scratch healing in comparison with Cur-PLA scaffolds [164]. Cur incorporation in poly(ethylene glycol) (PEG)-polycaprolactone (PCL) co-polymeric fibre mats showed antioxidant efficacy in primary dermal fibroblasts in vitro [134] and an advantage in promoting wound healing in vivo was reported. Cur nanoformulation was loaded into modified-chitosan film, in order to evaluate wound healing in animal models. A faster wound reduction and better healing was observed in comparison with the unloaded film [116].

Cur has also been investigated in combination with other natural antioxidants, such as *Aloe vera* (AV). Confident in their endorsing antioxidant properties, pectin–gelatin matrices were associated with AV and/or Cur [186]. The cytocompatibility, evaluated using 3T3 fibroblasts, as well as in vivo wound healing results, demonstrated that the AV-matrix was better than the Cur-matrix. In particular, AV exhibited an anti-inflammatory effect. AV had a major antimicrobial activity and enhanced apoptosis in vitro in comparison with Cur. The fact that AV seemed more efficient for in vivo healing than Cur was reinforced by a study where the usage of polymethacrylic acid silver nanogel matrix was combined with AV or Cur for coating of wound dressing [230]. AV-scaffold was firstly identified [182] as a better inducer of healing, in comparison with the poly(lactic-co-glycolic acid) (PLGA) scaffold alone. In vivo, a higher water uptake was shown for lipid nanoparticles (LNP)-PLGA-AV membranes that attracted a minor number of macrophages in wounds [183]. Seeking enhanced wound healing activity, the PCL-AV scaffold was improved by the loading of tetracycline hydrochloride [147]; in particular, tensile strength, dermal fibroblasts activity, and broad antibacterial efficacy were superior than in PCL-AV-Cur scaffolds. One interesting finding is that AV can modulate collagen production during all of the phases of wound healing. This aspect was recently investigated by evaluating the effect of AV-collagen gel on cell adhesion markers [231]. Other biomaterials investigated include decellularized bone matrix as a dressing with adipose stem cell-laden AV hydrogel. Overall, during the comparison between the experimental groups, AV modulated the expression of interleukin-1 (IL-1), transforming growth factor beta (TGF-β), and basic fibroblast growth factor (bFGF), concluding that the developed composite accelerated rabbit burn wound healing [224]. By preparing a novel hydrogel from human amnion combined with the AV extract that was able in vitro to promote HFF1 fibroblasts growth and HaCaT scratch closure, in vivo AV improved rat wound recovery rates and contraction [198]. The combined action of AV and amniotic membrane demonstrated beneficial epidermis regeneration and an increased number of blood vessels. The plasticization of ALG with PEG-methyl ether methacrylate was associated with both AV and *Moringa oleifera* (MO) to construct a porous dressing scaffold. Just as it was built, AV was responsible for an increased water uptake and MO was responsible for specific antimicrobial activity. AV together with MO enhanced total antioxidant activity and nitric oxide scavenging capacity was achieved. In addition, the phenolic components present in AV and MO cooperatively helped CCD-1112SK skin fibroblasts to faster proliferate in comparison with the scaffold without plant extract [109]. A scaffold used in autologous skin graft was based on collagen type I and modified with microparticles of gelatine-collagen type I, in order to evaluate the introduction of AV. The results were again not significant in terms of in vivo healing; however, as confirmation of the cross-linking issue, the observed decreased biodegradability of the material was associated with decreased in vitro enzyme degradation, that is, collagenase [148].

Of course, there are several other examples of natural antioxidants that have been explored extensively in wound healing apart from Cur and AV. Hydroglycolic extract of the *Calendula officinalis* (CO) flower was trialled instead of AV in one preclinical study [151], but it did not demonstrate the loaded scaffold as superior grafting material, likely because the extract concentration chosen for loading increased scaffold cross-linking. HPG was used to obtain electrospun nanofibers containing bioactive preparations of CO for optimal anti-inflammatory wound-healing membranes development. The bioactive wound dressing material was suitable for the culture of CHO hamster epithelial cells, bioadhesive, and it was not irritant and re-epithelizing in different animal models [190]. Cellulose acetate and zein nanofiber membranes were loaded with sesamol, a lignan, to investigate the effect on impaired wound healing frequently occurring in patients with diabetes. The study showed how the sesamol addition accelerated wound closure and enhanced in vivo transcriptional levels of TGF-β and alpha smooth muscle actin (α-SMA). The decreased levels of IL-1, tumor necrosis factor alpha (TNF-α), and nitric oxide synthases (NOS) were only owing to nanofiber intervention [232]. The PCL nanofibers loaded with Bixin, an antioxidant carotenoid that can be extracted from *Bixa orellana L*., ameliorated mice diabetic wound repair. The hydrophobicity of the antioxidant decreased pre-adipocite fibroblasts adhesion and proliferation [233], excluding hypertrofic scar formation. Of note, astaxanthin (AST) was loaded in collagen gel film, displaying good water absorption and good wound contraction. The remarkable wound healing activity (re-epithelization, fibroblast proliferation, and new ECM quality) in rats was faster than the one related to collagen hydrogel incorporating gentamicin instead of AST [161]. A gelatin-based hydrogel was tested in wound healing in vivo model because of good mechanical properties, swelling ability, and antibacterial effect, which was enhanced by the controlled release of the antioxidant ostholamide. The rats receiving ostholamide released by the fibrous scaffold exhibited enhanced healing when compared with the untreated control [158]. The cumulative effect of silver and plumbagin for antibacterial activity was observed in a collagen cross-linked scaffold for wound dressing application [159]. The composite showed cross-linking efficiency and water uptake similar to glutheraldehyde treatment, while the biocompatibility with 3T6 mouse fibroblasts was confirmed. The plumbagin caged silver nanoparticles composite was superior to the native collagen for the in vivo healing and more antibacterial than the plumbagin extract alone. A mixture of hyaluronic acid (HA) conjugated with EGCG and tyramine were explored as anti-inflammatory adhesive biomaterials for potential clinical application [145]. Rheological analysis, gel degradation, biocompatibility, and inflammation in vitro and in vivo were all investigated. The EGCG amount correlated with a decrease in TNF-α expression level in lipopolysaccharides (LPS)-stimulated RAW264.7 cells. Reduction of other pro-inflammatory factors in animal model serum and antibacterial behaviour was also shown. The healing in a rat skin incision model was more efficient in comparison with commercial medical grade adhesives. The presence of collagen into SF-fenugreek nanofibers reduced inflammation and accelerated epithelialization during wound healing, in comparison with the other tested sample group [117]. Soybean-modified poliammide 6 (PA-6) nanofibrillar matrices were explored for their potential as topical dermal covering for skin regeneration [167]. Qu and immobilized silver (Ag) were utilised as coating for functionalized polydimethylsiloxane, with a polydopamine layer as an intermediate layer. The combination used as coating for cotton gauze wound healing was effective for days in terms of antioxidant activity and showed a synergistic antimicrobial activity, while showing reduced inflammation [125]. As polydopamine can coat many surfaces, this approach may serve for functionalization of natural polymers too. Films based on CH-ALG were loaded with both thymol and β-Carotene to produce new wound dressings [234], unfortunately without deciphering the biological advantage of the antioxidants. A multiresponsive catechol-Fe^3+^ coordination hydrogel (MICH) was designed by assembling together HA, dopamine, β-ciclodestrin, and vitamin E [142]. This biomaterial was proven to supply in vitro antioxidant activities in different chemical models for different cell compartments; then, it decreased apoptosis and increased intracellular (NIH-3T3) ROS removal. In vivo, MICH attenuated oxidative stress, suppressed inflammation (TNF-α and count of white blood cells) and lipid peroxidation, increased re-epithelization, promoted neoangiogenesis (vascular endothelial growth factor (VEGF) analysis), and restored dermal normal collagen architecture. Collectively, these data present an injectable in situ gelling antioxidant biomaterial, worthy by combining scavenging and upregulation of endogenous enzymes (superoxide dismutase (SOD), glutathione peroxidase (GSH-Px), and catalase (CAT)). Moreover, by providing an optimal hydration to the wound it potentially will prevent wound necrosis. Very recently, a cellulose acetate-gelatin mat has incorporated berberine, a natural alkaloid. Water uptake, L929 murine fibroblastic cells proliferation, and antimicrobial activity were enhanced by the antioxidant that improved wound healing in in vivo diabetic models, as well as by angiogenesis [162].

The above studies describing potential application in wound healing of functionalized biomaterials are summarized in Table 2.

### 5.2. Bone Tissue Engineering

Owing to people aging, there is an increasing demand for bone autografts, but the procedure is limited primarily by surgical risk and the need for donor tissue [235]. Bone tissue engineering is an alternative intervention and the search for a new biomaterial with improved osteoconductivity, osteoinductive, osteogenic, and bioactivity is extensive [236]. Again, natural, synthetic, and composite biomaterials have been explored with antioxidants. A mesh of electrospun polydioxanone (PDX) was blended with a mixture of acemannan [237]/glucomannan from AV. Multicellular responses in vitro were assessed and showed enhanced 3T3 fibroblast proliferation and L929 fibroblast migration, spreading of Eahy926 endothelial cells, activation activity of RAW 264.7 macrophages, and mineralization by SaOS2 pre-osteoblasts. In comparison with similar PDX meshes blended with poly(hydroxybutyrate-co-hydroxyvalerate) (PHPV) and/or hydroxyapatite (Hap), the AV-containing material showed a better in vivo bioperformance for skeletal regeneration in the rat model, highlighting a minor foreign body response and angiogenesis [199]. On the other hand, AV gel-blended PHBV scaffold was identified as a promising osteoinductive suitable material for bone bioengineering, thanks to feasible differentiating cultures of iPSCs on it [204]. Gelatin was also chemically enriched with EGCG, with in vitro culture of mouse stem cells interestingly expressing osteogenic properties at day 1, in comparison with uncombined presence of the scaffold and of EGCG. In particular, the controlled release was evident in the gel-cell interaction and scaffold biodegradation allowed the release during osteogenesis in vivo [178]. Similarly, a sponge-like scaffold with different cross-linking chemistry was shown to induce osteogenesis of UMR106 osteoblasts in vitro and in vivo in calvarian defect as a result of EGCG action [207]. Gallic acid, another antioxidant, was incorporated in titanium substrate in order to deliver it for potential bone contact application [238]. The possibility to functionalize hydroxyapatite with Qu by different chemical syntheses for bone regeneration was explored in a triple co-culture model comprising MG63 osteoblast-like in contact with the biomaterial, osteoclast precursors 2T-110, and HUVECs [201]. As antioxidant activity was provided to the scaffold and a positive influence on bone repair microenvironment was shown, a scaffold of Hap functionalized with anti-osteoporotic drug, alendronate, was loaded with Qu. The new scaffold was evaluated in vitro for properties modulating bone loss and preventing related diseases [202]. Interestingly, the biomaterial promoted osteoblast proliferation and differentiation and inhibited osteoclast viability in oxidative stress treated co-culture; in parallel, TNF-α level in culture medium was decreased and Qu was the most antioxidant factor. A SF/Hap scaffold incorporating Qu for bone tissue-engineered graft investigation was based on evaluation of rat bone marrow (BM)-mesenchymal/stromal stem cells (MSCs). The biomaterial antioxidant activity was not measured, but the loading of appropriate Qu concentrations (0.03 wt %) was osteoinductive both in vitro and in vivo [239]. In MC3T3-E1 mouse osteoblast precursor model, controlled loading of Cur in PCL nanofibers enhanced molecular and histochemical markers of osteogenesis compared with the neat polymer, suggesting wider benefits of this antioxidant in bone tissue engineering [175].

A gelatin-based Hap foam [146] was functionalized with soybean extract, after previous characterization in SaOS cells. Then, the soybean biomaterial was implanted and it reduced the degradation rate and increased biomechanical strength, with denser bone formation in rabbit defect compared with control foams [240]. One hypothesis is that isoflavones released in soybean, like genistin, could act as a bone influencer. Soybean-oil-based scaffolds have been utilized to mimic a 3D microenvironment, for example, for osteogenic activity [241], but they were chosen because of their suitability for biocompatible polymer production, rather than the antioxidant properties. Both *Equisetum arvense* hydroethanolic extract and nano-(Hap) were loaded into PLA meshes, favoured proliferation, and increasing osteogenesis of AD-MSCs in comparison with PLA with nano-Hap only [156]. *Broussonetia kazinoki* plant extract modified an SF-based scaffold. The developed scaffolds were evaluated in rat BM-MSCs in vitro and in two in vivo murine models of bone defects [242]. The downregulation of the messenger ribonucleic acid (mRNA) level of pro-inflammatory TNF-α and cyclooxygenase-2 (COX-2) together with the upregulation of the pro-osteogenic markers were observed. The expected antioxidant extract of *Cissus quadrangularis* [243,244] was investigated on CH based-scaffolds to demonstrate bone formation in different in vitro cell models capable of osteogenic activity [245].

The above studies describing potential application in bone regenerative medicine of functionalized biomaterials are summarized in Table 2.

### 5.3. Cardiovascular Tissue Engineering

PVA-dextran hydrogel patches releasing astaxanthin (AST) and delivering its scavenging activity were in vitro compatible in HUVEC and 3T3 fibroblast cell models; this hydrogel was also suitable for in vivo suture and subcutaneous implantation [189]. These results are the basis for future evaluation of hydrogel effect on myocardial tissue recovery. Nanofibrous PLA-PCL-SF scaffolds including AV showed improved growth and differentiation of murine cardiomyocytes [205]. A modified surface of Mg-Zn-Y-Nd alloy, coated with TA, retained great scavenging activity even after some recycling. In addition, the platelet-repellent capability and subcutaneous implantation suggested similar application for vascular stent biomaterials [128]. Cur was included to prepare capped gold loaded PLGA nanoparticles. The animal models preserved their cardiac functions; Cur improved the stability of the particles; and Cur-PLGA nanoparticles played an important role in protection from cardiac hypertrophy, toxicity, and heart failure [154]. Vitamin E can modify the interaction between its functionalization products, such as PLA-derived polymers, and the host cells. For example, it reduced osteoblastic adhesion in vitro [166] and smooth muscle cell proliferation, thus suggesting a lower risk of in-stent restenosis [246]. In addition, bacterial [247] adhesion to this polymer was limited [222]. Chitosan-loaded with liposomes of vitamin E could be an appropriate sol-to-gel injectable material for myocardial repair. The survival of cardiomyocytes and resistance to oxidative stress environment were demonstrated [196]. The functionalization of electrospun gelatin-PCL nanofibers with salvianolic acid B and magnesium L-ascorbic acid 2 phosphate enhanced the physico-chemical properties of the scaffolds mimicking cardiac extracellular matrix. Cardiomyocytes proliferation and cardiac marker expression analysis demonstrated the potential for cardiac regeneration of that biomaterial [203]. The above studies describing potential application in cardiovascular regenerative medicine of functionalized biomaterials are summarized in Table 2.

### 5.4. Neural Tissue Engineering and Other Tissue Application

The human nervous system is especially vulnerable to oxidant stress-related injury owing to disease or aging [248] and has a low regeneration profile [249]. Prior to tissue engineering, surgical sutures were the only possible treatment for nerve injury [249]. The regeneration and tissue engineering of peripheral nerve grafts using a conductive biomaterial would overcome such issues. However, there is the problem of attaining adequate therapeutic delivery to the central and peripheral nervous system [139], coupled with the intrinsic short half-life of traditional compounds [250]. As such, the literature about biomaterial functionalized with natural antioxidant for nervous system therapy is relatively sparse. Specific vitamins and β-carotene regulate production of melanin, an endogenous antioxidant in skin [251] that exhibits unique electrical properties [252]. Melanin introduction in aligned SF fibers promoted neuronal growth and neurogenic activity in differentiating SH-SY5Y neuronal-like model. In addition, a scavenging activity regulated by the heteropolymer was detected [123]. As said for AV, other natural antioxidants were combined in the form of a polymer source of antioxidants with the biomaterials. Lignin nanoparticles combined with PCL fibers increased water uptake and biomechanical resistance in comparison with control samples. The lignin biomaterial showed great biocompatibility for rat pheochromocytoma PC12 cell line and human adipose derived stem cells. The neuronal differentiation induction of the two cellular models demonstrated that lignin content promoted differentiation and a particular amount (15%) favoured in vivo nerve tissue regeneration [100]. PCL nanofiber scaffold with lignin was also investigated for the biocompatibility with both human BM-MSCs (as common cells used in nerve tissue engineering) and rat Schwann cells. Free radical inhibition was associated with the biomaterial. The cultured Schwann cells grew on lignin PCL electrospun fibers; they were still viable after induced oxidative stress and up-regulated myelin expression. Moreover, primary neurons were able to grow on the developed biomaterial [100]. Other studies, reported below, pointed out the possibility to apply natural antioxidants and biomaterials for new tissue engineering protocols for tissues like cartilage and endometrium (Table 2). After simply mixing oxidized HA with resveratrol solution to make a cross-linked injectable hydrogel, the new biomaterial was evaluated in chondrocyte in vitro model. Here, resveratrol showed upregulation of chondrogenesis markers in the presence of LPS, together with downregulation of interleukin 1 beta (IL-1b) and some MMP [160]. A composite of sodium ALG and AV was used as bioink hydrogel for printing of endometrial MSCs onto a PCL 3D-printed mesh [223]. Following in vitro biocompatibility, an in vivo study was conducted to evaluate its potential in the treatment of patients with pelvic organ prolapse. In fact, AV-containing hydrogel could enhance the immunomodulatory effect of the stem cells and suppressed the foreign body response to the implant. In particular, AV-ALG reduced M1 macrophage infiltration with respect to the pure hydrogel. The above studies describing potential application in neural regeneration research of functionalized biomaterials are summarized in Table 2.

## 6. Conclusions

**Figure 6:**
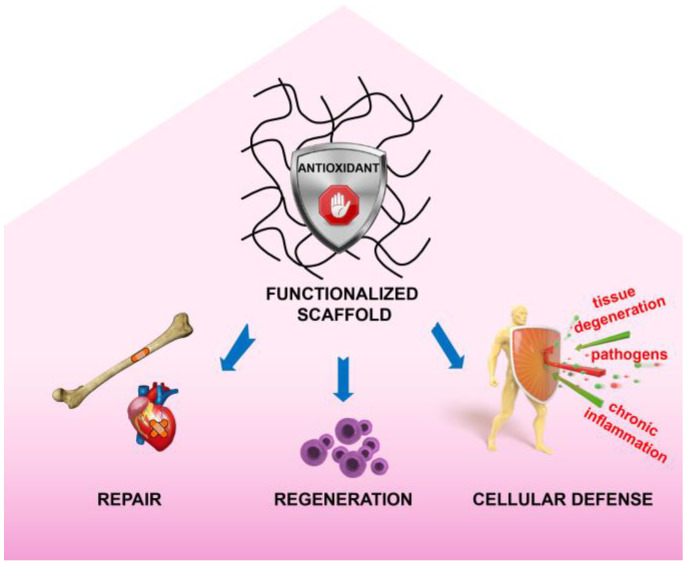
Schematic representation of the beneficial effects (repair, regeneration, and cellular defense) mediated by biomaterials incorporating natural antioxidants and demonstrated by their experimental application in tissue engineering and other therapeutic interventions.

New methods to specifically deliver regenerative trophic factors and to carry out immunotolerance are requested for the ultimate clinical success of implanted graft based on biomaterials. In nature, there is the availability of a plethora of antioxidants compounds that are able to maintain the redox balance. Many of these compounds have just the capacity of neutralizing pro-oxidant species, however, some of them are indirect natural antioxidants that can enhance endogenous antioxidant defences; prevent oxidative stress; and, intriguingly, support the functional survival of a bioengineered tissue. This review has collected a panel of studies about biomaterials developed and functionalized with different antioxidants derived from living organisms. Many natural antioxidants have the potential to be a source of pro-regenerative stimuli. Interestingly, the data presented in Table 2 highlighted that the majority of the studies investigating osteogenesis and bone regeneration and differentiation used polyphenols as antioxidant functionalizing agents. The majority of the analysed antioxidants showed an antimicrobial effect (Table 1) and many are antagonists of mediators of inflammation (Table 1 and Table 2). Using such natural antioxidants for functionalization of old and new biomaterials could provide a clinical advance in terms of repair, regeneration, and cellular defence, which are effective in the absence of oxidative stress (Figure 6). Antioxidants can be presented to both transplanted budding cells and pre-existing recovering cells integrated as part of the biomaterial scaffold. Innovative biomaterials can be utilized as scaffolding blocks with human stem cells in emerging bioprinting application, including drug screening and disease modelling.

A clinical challenge is to achieve transplants that integrate advanced functional biomaterials with stem cells and biological regulators (such as antimicrobial, immunomodulatory, and trophic compounds) [253]. Redox equilibrium can be a key player in modulating these regulators; namely, the antioxidants can play a central role as immunomodulators [254]. A promising assumption in post-operation could be derived from an augmented bioavailability in the human body of natural products in association with standard pharmaceutical therapy. That combined utilization of antioxidant mix is an approach to sustain oxidative balance in tissues more powerfully than a single agent performance. The concentration of antioxidant to deliver in vivo, as the most in vitro literature explains, is critical to address its beneficial role. A deeper insight and further research are needed for complex antioxidant formulas derived from some natural product comprising a lot of bioactive compounds, for avoiding the risk of unwelcome consequences. The methods for the extraction of herb antioxidants will benefit from standardization prior to the inclusion in tissue engineering material; as an alternative and opportune action, complex mixture and natural extracts should be selected and isolated to address a specific efficacy. Finally, this field of investigation will encourage the repurposing of antioxidants for human tissue repair and regeneration in prospective clinical trials, and highlights the powerful role of redox modulation in a therapeutic window.

## Figures and Tables

**Table 1:** Studies demonstrating the advantageous properties provided by the incorporation of specific natural antioxidants into various biomaterials.

Property	Antioxidant	Biomaterial (Scaffold)
Scavenging activity	*Aloe vera* (extract) [109] Astaxanthin [110] Caffeic acid [111] Carvacrol [112] Catechin [113] Chlorogenic acid [114] Cinnamon E.O. [115] Curcumin [116] Eugenol [112] Fenugreek (seed absolut) [117] Gallic acid [118] Hydrolyzed spent coffee grounds [119] Lemongrass E.O. [120] Mango (extract) [121,122] Melanin [123] *Moringa oleifera* (extract) [109] Quercetin [113,124,125] Rosmarinic acid [126] Rosemary (extract) [127] Tannic acid [128,129] *Teucrium polium* (extract) [130] Thyme (polyphenol extract) [131] Thymol [132] Vitamin C [133]	Cellulose-PEG [120] CH [112,113,122,132] CH-derivates [118,131] CH-gum [115] HA [129] Halloyste [124] Magnesium alloy [128] P(3HB-co-3HV) [133] PDMS [125] PE [119] PEG–ALG [109] PEG-PCL [134] PLA –PEG [130] PLA [127] PVP [121] SF -collagen [117] SF [123] Silica [111,126] Silica-PEG [114]
Indirect antioxidant effects (including cytoprotection)	Citric acid [135] Curcumin [136,137] Cystamine [73] Gallic acid [138] Hydrolyzed spent coffee grounds [119] Lignin (alkali) [139] Quercetin [136] Rapeseed flower (pollen extract) [140] Vitamin C [141] Vitamin E [142] White tea (leaves extract) [143]	CH [138] CH-derivates [116] Hyaluronic acid-β-cyclodextrin [142] Montmorillonite [143] PBAE [136] PCL [137,139] PE [119] PU [141] PVA [135]
Biodegradation modulation	*Aloe vera* [144] EGCG [145] Soybean (extract) [146] Tannic acid [129] Vitamin C [133]	Gelatin-Hap [146] HA [129,145] P(3HB-co-3HV) [133] SF [144]
Biomechanical stability	*Aloe vera* (extract) [147,148] Caffeic acid [149,150] *Calendula Officinalis* [151] Cinnamaldhyde [152] Citric acid [153] Curcumin [154] EGCG [155] Ellagic acid [155] *Equisetum arvense* (extract) [156] Grape (pomace) [157] Lignin (kraft) [100] Ostholamide [158] Plumbagin [159] Resveratrol [160] Soybean (extract) [146] Tannic acid [131,155] Vitamin C [133].	CH-Starch [131] Collagen [159] Collagen type I–Gelatin [148,151] Gelatin [153] Gelatin derivates-collagen [158] Gelatin-Hap [146] HA [160] P(3HB-co-3HV) [133] PCL [100,147] PCL-CH [149,150] PEG derivatives [155] PLA–derivates [152] PLA-Hap [156] PLGA [154] Porcine pericardium (decellularized) [157]
Hydrophilicity and wettability/swelling	*Aloe vera* (extract) [48,109] Astaxanthin [161] Berberine [162] Cinnamaldhyde [152] Curcumin [163,164] Lignin (kraft) [100] Ostholamide [158] Peppermint E.O. [165] Plumbagin [159] Rosemary (extract) [127] Sesamol [166] Soybean (oil) [167] *Thymus* (polyphenol extract) [168] *Zataria Multiflora* E.O. [169]	Cellulose acetate-gelatin [162] CH [168] Collagen [159,161] Gelatin derivates-collagen [158] HPG [164] PA-6 [167] PCL [48,100,165] PCL-gum [163] PEG-ALG [109] PLA [127,166] PLA-derivatives [152] PVA derivates [169]
Controlled release	Astaxanthin [170,171,172] Caffeic acid [173] Curcumin [116,174,175,176] Cystamine [73] EGCG [177,178] Morin [126] Ostholamide [158] Quercetin [157] Rosmarinic acid [179] Sage E.O. [180] Thymol [181] *Thymus* (polyphenol extract) [168]	ALG [170,177] CH-derivates [116,171] CH [168,180] Gelatin [178] Gelatin derivates-collagen [158] PBAE [73,176] PCL [175] Porcine pericardium (decellularized) [157] PVA [174] PVA derivates [181] Silica [126]
Biocompatibility	*Aloe Vera* (extract) [182,183,184,185,186] Astaxanthin [187,188,189] *Calendula Officinalis* (extract) [190,191,192] Cinnamon, E.O. [193] Curcumin [116,134,154,163,164,186] Lemongrass E.O. [193] Peppermint E.O. [193] Plumbagin [159] Rosmarinic acid [194] Soybean (oil) [167] Tannic acid [195] Tyramine [145] Vitamin E [196]	Cellulose acetate [193] CH [184,194,196] CH-derivates [116] Collagen [159] Gelatin [186] HA [145] HPG [164,190] PA-6 [167] PCL [185] PCL-gum [163] PCL-gum [191,192] PEG-PCL [134] PLGA [154,182,183,187,188] Poly-tannic acid [195] PVA derivatives [189]
Cell proliferation	*Aloe Vera* (extract) [48,109,144,197,198,199] Caffeic acid [149,200] Curcumin [154] Lignin (alkali) [139] *Moringa oleifera* (extract) [109] p-coumaric acid [200] Quercetin [201,202] Salvianolic acid B [203] Thymol [132] Vitamin B6 [200] Vitamin C (Magnesium phosphate) [203]	Amnion (decellularized hydrogel) [198] CH [132] Hap [201,202] PCL [48,139] PCL-gelatin [203] PDX [199] PEG-ALG [109] PLA [200] PLGA [154] SF [144] SF-PVA [197]
Cell differentiation	*Aloe vera* (extract) [204,205] Astaxanthin [206] EGCG [178,207] Lignin (alkali) [139] (kraft) [100] Melanin [123] Quercetin [201] Salvianolic acid B [203] Soybean lecithin [208] Vitamin C (Magnesium phosphate) [203] Vitamin C [141]	Gelatin [178,207] Gelatin derivatives [206] Hap [201] PCL [100,139] PCL-gelatin [203] PHBV [204] PLA-PCL-SF [205] PLGA [208] PU [141] SF [123]
Antimicrobial activity	*Aloe vera (extract)* [209] Berberine [162] Caffeic acid [149] *Calendula Officinalis* (extract) [192,210] Carvacrol [112,211] Chlorogenic acid [114] Cinnamaldhyde [152] Cinnamon E.O. [193,212,213] Curcumin [174] Eugenol [112] Lemongrass E.O. [193] Mango (silver particles) [214] *Moringa Oleifera (extract)* [109] Ostholamide [158] Peppermint E.O. [165,193] Plumbagin [159] *Punica granatum* (peel extract) [215] Quercetin [125,201] Rosemary (extract) [127] Rosmarinic acid [216] Sage E.O. [180] *Teucrium polium* (extract) [130] Thyme and Oregano E.O. [217,218,219] Thymol [132,220,221] Vitamin E [222] *Zataria Multiflora* E.O. [169]	ALG [211] Cellulose acetate [193,221] Cellulose acetate-gelatin [162] Cellulose-PEG [120] CH [112,210,220] CH-derivatives [215,218] CH-gelatin [219] Collagen [159] Gelatin derivates-collagen [158] Gelatin-gum [216] Hap [201] Ionomer cement [214] PCL [165] PCL-CH [149] PCL-gum [191,192] PDMS [125] PEG-ALG [109] PLA [127] PLA-CH [212,213] PLA-derivatives [152,222] PLA-PEG [130] PVA [174] PVA derivates [169] PVP [209] Silica-PEG [114] β-cyclodextrin [217]
Immunomodulation and anti-inflammatory effect	*Aloe vera* (extract) [223,224] Citric acid [135] Curcumin [154] EGCG [145] Quercetin [202] Resveratrol [225,226] Soybean [227,228]	ALG [223] CH [227,228] Cow bone matrix (decellularized) [224] HA [145] Hap [202] PLGA [154] PVA [135] SF [225,226]

ALG, alginate; CH, chitosan; E.O., essential oil; HA, hyaluronic acid; Hap, hydroxyapatite; P(3HB-co-3HV), poly(3-hydroxybutyrate-co-3-hydroxyvalerate); PBAE, poly(β-amino ester); PCL polycaprolactone; PDMS, polydimethylsiloxane: PDX, polydioxanone; PE, polyethylene, PEG, polyethylene glycol; PHBV, poly(3-hydroxybutyrate-co-3-hydroxyvalerate); PLA, polylactic acid; PLGA, poly(lactic-co-glycolic acid); PU, polyuretane; PVA, polyvinyl alcohol; PVP, polyvinylpyrrolidone; SF, silk fibroin.

**Table 2:** Studies demonstrating the potential therapeutic application of biomaterials including natural antioxidants.

Therapeutic Application	Antioxidant	Biomaterial
Wound healing	*Aloe vera* [147,183,186,198,224,230] Astaxanthin [161] Berberine [162] Bixin [233] *Calendula officinalis* [190] Curcumin [134,147,164,186,230,233] Dopamine [125,142] EGCG [145] *Moringa oleifera (extract)* [109] Ostholamide [158] Quercetin [125] Sesamol [232] Soybean (extract) [167] Thymol [234] Tyramine [145] Vitamin E [142] β-Carotene [234]	ALG derivatives [109] Amnion (decellularized hydrogel) [198] Bone matrix (decellularized) [224] .Cellulose acetate–gelatin [162] Cellulose acetate derivatives [232] CH derivatives [116] CH-ALG [234] Collagen [161] Gelatin derivative [186] Gelatin derivatives-collagen [158] HA [145] HA-β-cyclodextrin [142] HPG [190] PA-6 [167] PCL [148,236] PDMS [125] PEG-PCL [134] PLA-HPG [164] PLGA derivatives [183] PMAA [230]
Bone defects	*Aloe vera* (acemannan/glucomannan) [199] (extract) [204] *Broussonetia kazinoki* (extract) [242] *Cissus quadrangularis* (extract) [245] Curcumin [175] EGCG [178,207] *Equisetum arvense* (extract) [156] Fenugreek (seed absolut) [117] Quercetin [204,205,243] Soybean (extract) [240] Soybean (oil) [241]	CH derivatives [245] Gelatin [178,207] Gelatin-Hap [240] Hap [201,202] PCL [175] PDX derivates [199] PHBV [204] PLA-Hap [156] PS [241] SF [242] SF-collagen [117] SF-Hap [239]
Cardiovascular diseases	*Aloe Vera* [205] Astaxanthin [189] Curcumin [154] Salvianolic acid B [203] Tannic acid [128] Vitamin C (Magnesium phosphate) [203] Vitamin E [196,246]	CH [196] Magnesium alloy [128] PCL-gelatin [203] PLA [246] PLA-PCL-SF [205] PLGA [154] PVA derivatives [189]
Neurological disorders	Lignin (alkali) [139] (kraft) ([100]) Melanin [123]	PCL [100,139] SF [123]
Other tissue disorders	*Aloe vera* [223] Resveratrol [160]	ALG [223] HA [160]

ALG, alginate; CH, chitosan; HA, hyaluronic acid; Hap, hydroxyapatite; P(3HB-co-3HV), poly(3-hydroxybutyrate-co-3-hydroxyvalerate); PA-6, polyammide 6; PCL, polycaprolactone; PDMS, polydimethylsiloxane: PDX, polydioxanone; PE, polyethylene, PEG, polyethylene glycol; PHBV, poly(3-hydroxybutyrate-co-3-hydroxyvalerate); PLA, polylactic acid; PLGA, poly(lactic-co-glycolic acid); PMAA, polymethacrylic acid; PS, polystyrene; PVA, polyvinyl alcohol; PVP, polyvinylpyrrolidone; SF, silk fibroin.

## Abbreviations

3T3	Swiss albino mouse fibroblast (cell line clone)
3T6	Swiss albino mouse fibroblasts (cell line clone)
α-SMA	alpha smooth muscle actin
ALG	alginate
AST	astaxanthin
AV	Aloe vera
ARE	antioxidant response element
bFGF	basic fibroblast growth factor
AD-MSC (s)	adipose (derived) mesenchymal/stromal stem cell(s)
BM-MSC (s)	bone marrow mesenchymal/stromal stem cell(s)
CAT	catalase
CH	chitosan
CHO	Chinese hamster ovary (cells)
CO	Calendula Officinalis
COX-2	cyclooxygenase-2
Cur	curcumin
Eahy926	human umbilical vein endothelial cells (hybrid cell line)
EGCG	epigallocatechin gallate
E.O.	essential oil
ECM	extra cellular matrix
GF	growth factors
GMP	good manufacturing practices
GSH-Px	glutathione peroxidase
GVHD	graft versus host disease
HA	hyaluronic acid
Hap	hydroxyapatite
HFF1	human foreskin fibroblast (cell line)
HUVEC	human umbilical vein endothelial cells
IL-1	interleukin-1
IL-1b	interleukin 1 beta
iPSC	induced pluripotent stem cells
Keap1	Kelch-like ECH-associated protein 1
L929	mouse subcutaneous connective tissue fibroblast (cell line)
LPS	lipopolysaccharides
M1	Th1-response macrophage phenotype (classically activated)
M2	Th2-response macrophage phenotype (alternatively activated)
MC3T3-E1	mouse preosteoblast (cell line)
MG63	human osteosarcoma (cell line)
MICH	multiresponsive catechol-Fe3+ coordination hydrogel
MO	Moringa oleifera
MMP	matrix metalloproteinases
mRNA	messenger ribonucleic acid
MSC	mesenchymal stem cells
NADPH	nicotinamide adenine dinucleotide phosphate
NF-kB	nuclear factor kappa-B
NIH3T3	mouse swiss NIH embryo
Nrf2	nuclear factor erythroid 2-related factor 2
NOS	nitric oxide synthases
NOX	NADPH oxidase
P(3HB-co-3HV)	poly(3-hydroxybutyrate-co-3- hydroxyvalerate)
PBAE	poly(β-amino ester)
PA	proanthocyanidins
PA-6	polyammide 6
PC12	pheochromocytoma (cell line)
PCL	polycaprolactone
PDMS	polydimethylsiloxane
PDX	polydioxanone
PE	polyethylene
PEG	polyethylene glycol
PHBV	poly(3-hydroxybutyrate-co-3-hydroxyvalerate)
PLA	polylactic acid
PLGA	poly(lactic-co-glycolic acid)
PMAA	polymethacrylic acid
PS	polystyrene
PU	polyuretane
PVA	polyvinyl alcohol
PVP	polyvinylpyrrolidone
Qu	quercetin
RA	rosmarinic acid
RAW264.7	Abelson murine leukemia virus transformed (cell line)
ROS	reactive oxygen species
SaOS2	human osteosarcoma (cell line)
SOD	superoxide dismutase
SF	silk fibroin
TA	tannic acid
TGF-β	transforming growth factor beta
TNF-α	tumor necrosis factor alpha
SHSY-5Y	human neuroblastoma (cell line)
UMR106	rat osteosarcoma (cell line)
VEGF	vascular endothelial growth factor
